# Privacy-preserving multimodal federated learning pipeline for cyber-resilient healthcare systems

**DOI:** 10.1371/journal.pone.0343669

**Published:** 2026-04-10

**Authors:** Md Iftekhar Monzur Tanvir, Habibor Rahman Rabby, Md Habibul Arif, Nusrat Yasmin Nadia, Kamruddin Nur

**Affiliations:** 1 Department of Information Technology, Washington University of Science and Technology, Alexandria, Virginia, United States of America; 2 Department of Computer Science, Campbellsville University, Louisville, Kentucky, United States of America; 3 Department of Information Technology, University of the Potomac, Potomac, Washington, D.C., United States of America; 4 Department of Computer Science, American International University-Bangladesh, Dhaka, Bangladesh; PLOS: Public Library of Science, UNITED STATES OF AMERICA

## Abstract

The integration of Internet of Things (IoT) devices and electronic medical records (EMRs) has transformed healthcare delivery but has also created new vulnerabilities to cyberattacks that threaten both data confidentiality and patient safety. Conventional centralized machine learning approaches for intrusion detection are impractical in this domain due to strict privacy regulations, heterogeneous data sources, and the risk of single points of failure. To address these challenges, we propose a secure distributed machine learning pipeline for cyber-resilient healthcare systems. The framework combines federated optimization with split learning for sensitive EMR data, robust aggregation to mitigate poisoned updates, and differential privacy with secure aggregation to protect against inference attacks. Multimodal fusion is enabled through temporal consistency regularization for IoT traffic and cross-layer contrastive alignment to link EMR representations, ensuring improved anomaly detection across diverse healthcare environments. Experiments conducted on representative IoT and EMR datasets demonstrate that the proposed pipeline achieves accuracy of 0.942 on IoT data, 0.931 on EMR data, and 0.953 in the combined setting, with corresponding F1-scores of 0.921, 0.908, and 0.932. Ranking metrics further confirm superiority with AUROC up to 0.961 and AUPRC up to 0.947, outperforming deep baselines by margins of +0.025 to +0.033. Robustness analysis shows graceful degradation under client poisoning (0.953→0.879 at 30% malicious clients) and resilience under severe communication constraints (accuracy 0.953→0.861 at 90% update sparsification). Detection latency is reduced to an average of 5.9 time steps, compared to 7.8 for the strongest deep baseline. These results highlight that secure distributed pipelines can deliver both strong detection capabilities and regulatory compliance, providing a practical path toward safeguarding next-generation healthcare infrastructures against evolving cyber threats.

## 1. Introduction

The digital transformation of healthcare systems has ushered in an era where patient care, medical decision-making, and operational efficiency increasingly rely on interconnected devices, electronic medical records (EMRs), and intelligent monitoring platforms [1,2]. While this integration of cyber and physical infrastructures improves accessibility and quality of healthcare, it also introduces unprecedented vulnerabilities. The proliferation of Internet of Things (IoT) medical devices and the extensive use of EMRs expose critical systems to cyber threats ranging from denial-of-service and data exfiltration to advanced persistent intrusions [3]. In this context, cyberattacks not only endanger data confidentiality but also pose direct risks to patient safety, making security and resilience indispensable pillars of modern healthcare infrastructure [4].

Against this backdrop, a specific research challenge emerges: how can machine learning pipelines be designed to simultaneously provide accurate anomaly detection, safeguard patient privacy, and remain resilient under adversarial and distributed conditions? Traditional centralized machine learning approaches fall short because they require aggregating sensitive data across institutions, which is infeasible due to strict privacy regulations and logistical barriers [5,6]. Federated learning and related distributed methods offer promising directions, yet they remain vulnerable to poisoning, communication bottlenecks, and performance degradation when dealing with heterogeneous data sources such as IoT streams and EMRs [7].

The objective of this work is to design and evaluate a secure distributed machine learning pipeline that addresses these challenges by integrating multimodal data fusion, federated optimization, split learning for sensitive EMR data, and advanced privacy-preserving mechanisms [8]. The motivation is twofold: first, to improve the cyber-resilience of healthcare systems against malicious intrusions and, second, to establish a privacy-aware framework that complies with healthcare regulations while delivering state-of-the-art detection performance.

The significance of this research lies in its potential to redefine how healthcare systems defend against cyber threats. By unifying distributed optimization, robust aggregation, differential privacy, and multimodal data processing, the proposed framework demonstrates that it is possible to build secure and resilient pipelines without compromising the predictive power of deep learning [9]. This work contributes to the growing body of research at the intersection of cybersecurity, distributed learning, and healthcare informatics, addressing critical gaps in prior studies that often considered these aspects in isolation [10].

Methodologically, the framework combines IoT patient monitoring traffic and EMR datasets within a federated setting. Local clients train partial models on-site, leveraging temporal consistency regularization for IoT data and cross-layer contrastive alignment to fuse EMR features. Robust aggregation rules mitigate adversarial updates, while differential privacy noise addition and secure aggregation ensure compliance with privacy constraints. The overall architecture is evaluated using extensive metrics such as accuracy, precision, recall, F1-score, Area Under the Receiver Operating Characteristic curve (AUROC), and Area Under the Precision–Recall Curve (AUPRC), as well as resilience benchmarks under adversarial and resource-constrained settings.

The motivation behind this study arises from three critical challenges observed in real-world healthcare environments. First, cyber threats targeting hospitals and medical devices have increased significantly in recent years, with ransomware attacks and data manipulation incidents posing direct risks to patient safety and operational reliability [1,3]. Second, healthcare data are inherently distributed across hospitals, departments, and edge medical devices, making centralized machine learning approaches both impractical and non-compliant with privacy regulations such as HIPAA and GDPR [8,9]. Third, most existing intrusion detection research relies on a single data source—either network traffic or clinical records—failing to capture coordinated cross-layer attack patterns that span IoT devices and electronic medical records [11,12]. These challenges highlight the need for a secure learning pipeline that can jointly analyze multimodal healthcare data, operate in federated environments without sharing raw patient information, and remain resilient under adversarial threats.

### 1.1. Key contributions

To clearly articulate the contributions, the key highlights of this paper are summarized as follows:

- We propose a novel secure distributed machine learning pipeline that integrates federated optimization, split learning, and privacy-preserving mechanisms tailored for healthcare systems, achieving accuracy up to 0.953 and F1-score 0.932 on combined IoT and EMR data.
- We design multimodal fusion techniques combining IoT traffic and EMR data through temporal consistency regularization and cross-layer contrastive alignment, improving AUPRC from 0.919 (deep fusion baseline) to 0.947.
- We introduce resilience protocols against adversarial conditions such as poisoning attacks, communication dropouts, and latency constraints, maintaining accuracy of 0.879 with 30% malicious clients and 0.861 under 90% communication sparsification, while reducing IoT detection latency to 5.9 time steps (vs. 7.8 for CNN baseline).
- We conduct extensive experiments demonstrating superior detection performance, privacy guarantees, and resilience compared to conventional baselines, with consistent margins of +0.025 to +0.033 improvements in accuracy and F1-score across datasets.

The remainder of this paper is organized as follows. Section II reviews existing work on distributed learning and healthcare cybersecurity. Section III presents the proposed pipeline, including preprocessing, architecture, optimization, and privacy mechanisms. Section IV reports quantitative and qualitative results, along with ablation studies and resilience evaluations. Section V discusses the novelty, implications, limitations, and potential future directions of this research. Finally, Section VI concludes the paper with closing remarks.

## 2. Related work

The increasing reliance on digital infrastructures in healthcare has made the security and resilience of medical systems an important research focus. Early approaches to securing healthcare systems primarily relied on network intrusion detection and rule-based anomaly monitoring [13]. These traditional methods provided a foundation for identifying known attack signatures but struggled with zero-day threats, evolving adversarial strategies, and the growing scale of IoT-enabled medical devices [14]. With the emergence of machine learning, researchers began applying classification and clustering algorithms to detect malicious activity within medical data. These methods improved over heuristic systems but remained limited in scalability, adaptability, and robustness against sophisticated cyberattacks [15].

In recent years, deep learning-based intrusion detection frameworks have gained prominence. Convolutional, recurrent, and transformer-based architectures have been applied to analyze both temporal signals from patient monitoring devices and structured medical data [16]. While these models demonstrate strong predictive power, they often rely on centralized data aggregation, which is impractical in healthcare due to strict privacy regulations, data ownership concerns, and the risks associated with transferring sensitive patient information across institutions. Moreover, centralization introduces a single point of failure, increasing the vulnerability of healthcare systems to data breaches [17].

To overcome these challenges, distributed learning has emerged as a promising paradigm. Federated learning (FL) enables local model training on client devices while sharing only model updates with a central server [11]. This preserves data locality and mitigates privacy risks, making it highly suitable for healthcare systems where patient records are inherently distributed [18]. Various FL extensions address issues such as data heterogeneity, communication efficiency, and robustness against malicious clients. However, federated learning alone can still be susceptible to inference and poisoning attacks, highlighting the need for stronger privacy protection [12].

Recent work has extended FL toward cybersecurity and healthcare applications. Federated intrusion detection frameworks have been developed to detect large-scale cyber threats collaboratively without centralizing data [19]. Similarly, FL-based systems have been applied to defend against distributed denial-of-service (DDoS) attacks [20]. Privacy threats in FL are being mitigated using improved mechanisms such as randomized response-based differential privacy [21]. The practical feasibility of FL for real-world healthcare data has also been demonstrated in live hospital environments [22].

Complementary to FL, split learning enforces stricter privacy by partitioning models between client and server. Clients perform forward propagation up to an intermediate layer, share only the resulting activations (smashed data), and receive gradients during backpropagation. This ensures that raw patient data never leave the local site [23]. However, split learning introduces additional communication overhead and its efficiency depends on the chosen cut layer [24].

Another relevant research direction involves privacy-preserving mechanisms such as differential privacy and secure aggregation. Differential privacy introduces calibrated noise to model updates, reducing the risk of reconstructing sensitive data, while secure aggregation ensures that the server observes only the aggregated updates [25]. These mechanisms, when integrated into federated or split learning, form the foundation of privacy-aware distributed systems. Nevertheless, maintaining high model accuracy while enforcing privacy guarantees remains challenging, particularly under adversarial conditions [26].

The integration of multimodal data for anomaly detection has also attracted attention. IoT device streams and EMR records provide complementary signals that can improve the detection of cyber intrusions when analyzed jointly. Methods incorporating cross-modal attention, contrastive learning, and temporal regularization have been explored to align heterogeneous modalities [27]. However, many existing studies still rely on a single modality—either IoT or EMR—limiting their effectiveness in capturing cross-layer attack behavior [28].

Finally, research on adversarial resilience has advanced through robust aggregation, adversarial training, and client anomaly scoring [29,30]. These techniques mitigate poisoning, inversion, and dropout attacks, but most are studied in isolation. A comprehensive solution integrating privacy, robustness, multimodal learning, and distributed optimization remains underexplored.

Overall, existing research has achieved notable progress in securing healthcare data using machine learning. Yet, there is still a gap in unifying multimodal data fusion, distributed optimization, adversarial robustness, and privacy preservation within a single, cohesive framework. This study addresses that gap by proposing and evaluating a secure distributed learning pipeline specifically designed for healthcare cyber-resilience.

Table 1 summarizes key prior studies, highlighting their main focus, applied techniques, and existing limitations addressed by our proposed framework.

**Table 1:** Summary of key related studies in federated, privacy-preserving, and multimodal healthcare learning.

Study	Focus Area	Technique	Limitation / Gap
[14]	Secure IoT-based healthcare systems	Rule-based intrusion detection	Ineffective for zero-day and adaptive attacks
[16]	Deep learning for healthcare intrusion detection	CNN, RNN	Centralized training violates data privacy
[17]	IoT healthcare network protection	Hybrid DL-IDS	Limited scalability and privacy handling
[11]	Federated learning in healthcare	FL-based distributed optimization	Vulnerable to poisoning and inference attacks
[18]	Review of FL in medical data sharing	Cross-hospital training frameworks	Lacks multimodal fusion or adversarial robustness
[19]	Federated intrusion detection systems	FL for cyber threat detection	No privacy or cross-layer fusion considered
[20]	Federated defense against DDoS attacks	FL-based collaborative defense	No multimodal healthcare application
[21]	Differential privacy for FL	Randomized response-based DP	Trade-off between accuracy and privacy
[23]	Split learning for data confidentiality	Client-server model partitioning	High communication overhead
[24]	Split learning optimization	Intermediate-layer cut adaptation	Performance varies by cut position
[28]	Multimodal healthcare anomaly detection	Cross-modal fusion (IoT + EMR)	Lacks federated or privacy-preserving setup
[29]	Robust aggregation in FL	Byzantine-resilient rules	Evaluated only under synthetic attacks
[30]	Adversarially robust FL	Adversarial training	No healthcare deployment validation
[31]	Machine unlearning in FL	Privacy preservation via unlearning	Lacks integration with multimodal FL in healthcare

## 3. Methodology

**Fig 1:**
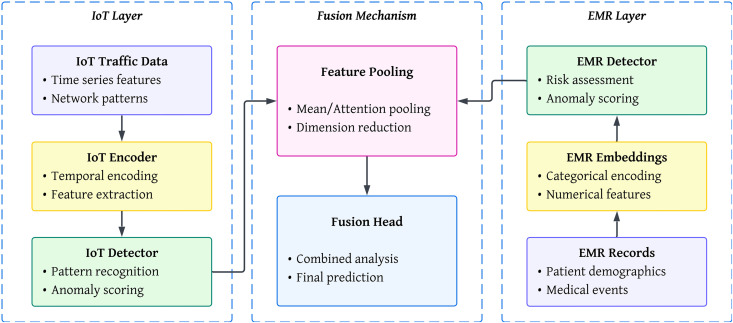
System architecture overview: IoT and EMR layers with encoders, detectors, fusion head, and final prediction path.

**Fig 2:**
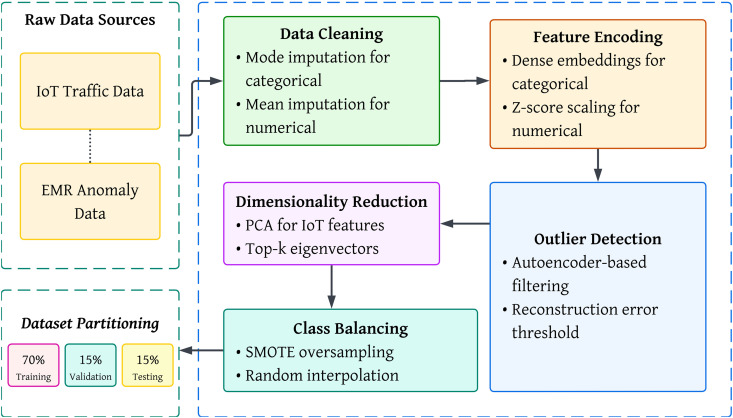
Preprocessing and learning pipeline: data cleaning, encoding, outlier filtering, dimensionality reduction, class balancing, and stratified splits.

This section describes our distributed learning pipeline for joint IoT and EMR cyber incident detection. We refer to the data preprocessing steps, problem setup, architectures, learning objectives, secure aggregation, robustness measures, and the training protocol. Fig 1 sketches the overall system architecture, while Fig 2 shows the end-to-end data pipeline that precedes learning.

### 3.1. Data preprocessing

The preprocessing stage was critical to ensure the IoT traffic data (Attack, environmentMonitoring, and patientMonitoring CSVs) and the EMR anomaly data (payload-combined CSV) were prepared for robust training in our secure distributed pipeline. Both datasets contained heterogeneous features, requiring systematic cleaning, transformation, and partitioning.

#### 3.1.1. Data cleaning and integration.

Each dataset was loaded into a structured pipeline. Missing values in categorical and numerical features were treated separately. For categorical attributes *x*_*c*_, the mode imputation was applied:


$${\hat{x}}_{c}=\arg\max_{v\in V}\;\text{count}(x_{c}=v),$$ (1)

where *V* is the set of possible values for feature *x*_*c*_. For numerical features *x*_*n*_, mean imputation was performed:


$${\hat{x}}_{n}=\frac{1}{N}\sum_{i=1}^{N}x_{n}^{\left(i\right)},$$ (2)

with *N* being the number of valid records.

After imputation, duplicate records were removed, and all datasets were integrated under a unified schema with labels y∈{0,1} indicating normal (0) or anomalous (1) activity.

#### 3.1.2. Feature Encoding and Normalization.

Categorical EMR features (e.g., race, gender, ethnicity) were converted into dense embeddings using a trainable embedding layer:


$${𝐳}_{c}={𝐖}_{e}\cdot one\_hot(x_{c}),$$ (3)

where ${𝐖}_{e}\in {\mathbb{R}}^{d\times |V|}$ is the embedding matrix and *d* is the embedding dimension.

Numerical features (e.g., packet lengths, TCP ports, patient age) were normalized using z-score scaling:


$$x_{n}^{\prime }=\frac{x_{n}-\mu }{\sigma },$$ (4)

where μ and σ are the mean and standard deviation of feature *x*_*n*_ across the training set. This ensured that all features contributed equally to the optimization process.

#### 3.1.3. Outlier detection and noise reduction.

To increase resilience against adversarial noise, we employed an autoencoder-based outlier filter. Given an input **x**, the autoencoder reconstructs $\hat{𝐱}$ and computes the reconstruction error:


$${ℒ}_{rec}=\|𝐱-\hat{𝐱}{\|}_{2}^{2}.$$ (5)

Samples with ${ℒ}_{rec}$ above a threshold τ were flagged as noisy and either corrected or discarded. This step reduced mislabeled or adversarially perturbed records from corrupting the training set.

#### 3.1.4. Dimensionality reduction.

High-dimensional IoT features (52 attributes per record) were projected into a lower-dimensional latent space using Principal Component Analysis (PCA). For each input vector $𝐱\in {\mathbb{R}}^{d}$, the transformation was:


$$𝐳={𝐔}^{T}𝐱,$$ (6)

where $𝐔\in {\mathbb{R}}^{d\times k}$ contains the top-*k* eigenvectors of the covariance matrix of the training data. This reduced feature redundancy and improved convergence stability.

#### 3.1.5. Data balancing.

Both datasets exhibited class imbalance, with normal samples dominating anomalous samples. To mitigate this, we applied Synthetic Minority Oversampling Technique (SMOTE). For each minority class sample **x**_*i*_, a synthetic point **x**_*new*_ was generated as:


$${𝐱}_{new}={𝐱}_{i}+\lambda \cdot ({𝐱}_{nn}-{𝐱}_{i}),$$ (7)

where **x**_*nn*_ is a randomly chosen nearest neighbor and λ~U(0,1) is a random scalar. This ensured a balanced training distribution and reduced classifier bias.

#### 3.1.6. Partitioning into training, validation, and test sets.

Finally, the integrated dataset was partitioned into training, validation, and test subsets with stratification to preserve the anomaly/normal ratio:


$${𝒟}_{train}:{𝒟}_{val}:{𝒟}_{test}=70:15:15.$$ (8)

Formally, if *N* is the total number of samples, then:


$$|{𝒟}_{train}|=0.7N,$$ (9)

$$|{𝒟}_{val}|=0.15N,$$ (10)

$$|{𝒟}_{test}|=0.15N.$$ (11)

The training set was used to optimize model parameters, the validation set to tune hyperparameters and prevent overfitting, and the test set to report final performance metrics.

### 3.2. Proposed framework

Before going through the framework, we would like to introduce some notation used in the paper. In this work, bold lowercase symbols (e.g., **x**) denote vectors, bold uppercase symbols denote matrices (e.g., **W**), and regular lowercase symbols denote scalars. The model is denoted by f(·;θ) with trainable parameters θ. Predicted anomaly scores are represented by $\hat{y}$, and ground truth labels by y∈{0,1}. Gradients are written as $\nabla_{\theta }$, expectation as 𝔼[·], and concatenation as concat(·). All symbols are defined upon first use for clarity and consistency.

#### 3.2.1. Problem setting and notation.

Let ${𝒟}^{iot}=\{({𝐱}_{i}^{iot},y_{i}^{iot}){\}}_{i=1}^{N_{iot}}$ denote records from the IoT ICU deployment (52 features per record after preprocessing), and ${𝒟}^{emr}=\{({𝐱}_{j}^{emr},y_{j}^{emr}){\}}_{j=1}^{N_{emr}}$ denote EMR payload records (mixed numerical/embedded categorical features). Labels are y∈{0,1} with 1 indicating an anomalous or malicious event. Fig 1 illustrates how these sources map to encoders, detectors, and the fusion head.

We partition data across *K* clients in a dual layer: (i) edge IoT clients ${𝒞}_{iot}$ (e.g., per bed or per device group), and (ii) hospital EMR clients ${𝒞}_{emr}$ (e.g., per department). Client *k* holds ${𝒟}_{k}$ with $n_{k}=|{𝒟}_{k}|$ and local parameters $\theta_{k}$. A central server maintains global parameters θ.

The global learning objective is the weighted empirical risk:


$$\min_{\theta }\;\;ℒ(\theta )=\sum_{k=1}^{K}\frac{n_{k}}{\sum_{\ell =1}^{K}n_{\ell }}\;{𝔼}_{(𝐱,y)\sim {𝒟}_{k}}[\ell (f(𝐱;\theta ),y)],$$ (12)

where f(·;θ) is the model and *ℓ* is a binary classification loss (defined below).

#### 3.2.2. Threat model.

We consider two adversarial surfaces: (i) *data poisoning* on a fraction *p* of clients, which alters local training samples or gradients, and (ii) *evasion* at inference time. We also assume an honest-but-curious server and enforce privacy with secure aggregation and differential privacy.

#### 3.2.3. Dual-Layer Architecture.

**IoT encoder and detector.** We model time-aware IoT records with a temporal encoder *g*_iot_ and a classifier *h*_iot_:


$${𝐳}^{iot}=g_{iot}({𝐱}^{iot};\theta_{iot})\in {\mathbb{R}}^{d_{iot}},$$ (13)

$${\hat{y}}^{iot}=\sigma (h_{iot}({𝐳}^{iot};\phi_{iot})),$$ (14)

where $\sigma (u)=1/(1+e^{-u})$. In practice, *g*_iot_ is a 1D CNN or GRU over a short sliding window, and *h*_iot_ is an MLP head.

**EMR encoder and detector.** We embed categorical EMR fields and concatenate with normalized numerics, then apply *g*_emr_ and *h*_emr_:


$${𝐳}^{emr}=g_{emr}({𝐱}^{emr};\theta_{emr})\in {\mathbb{R}}^{d_{emr}},$$ (15)

$${\hat{y}}^{emr}=\sigma (h_{emr}({𝐳}^{emr};\phi_{emr})).$$ (16)

**Cross-layer fusion (optional).** When IoT and EMR events co-occur within a time window Δt, we fuse representations:


$${𝐳}^{fuse}=concat\;(pool({𝐳}^{iot}),\;pool({𝐳}^{emr})),\;{\hat{y}}^{fuse}=\sigma (h_{fuse}({𝐳}^{fuse};\phi_{fuse})),$$ (17)

with pool a mean or attention pooling. This head is trained jointly with the layer-specific heads. The end-to-end steps that prepare inputs for these heads appear in Fig 2.

#### 3.2.4. Architectural details.

To ensure reproducibility, we specify the complete architecture for both IoT and EMR models, along with the fusion head. Each layer type, output shape, activation function, and parameter count is reported in Table 2. The IoT branch processes the 52-dimensional network traffic features, while the EMR branch processes 23 demographic and event fields after embedding and normalization. The fusion head integrates both representations when co-occurring events are present (see Fig 1).

**Table 2:** Architectural details of IoT branch, EMR branch, and Fusion head.

Component	Layer Type	Output Shape	Activation	Parameters
**IoT Branch**				
Input (IoT)	—	(52,)	—	—
Dense-1	Fully Connected (52 → 128)	(128,)	ReLU	52×128+128=6,784
Dense-2	Fully Connected (128 → 64)	(64,)	ReLU	128×64+64=8,256
Dropout	Dropout (*p* = 0.3)	(64,)	—	0
Dense-3	Fully Connected (64 → 32)	(32,)	ReLU	64×32+32=2,080
Output (IoT)	Fully Connected (32 → 1)	(1,)	Sigmoid	32×1+1=33
**EMR Branch**				
Input (EMR)	—	(23,)	—	—
Embedding (categorical fields)	Trainable Embedding (*d*_*e*_ = 16)	(≤ 10, 16)	—	≈ 5,000
Dense-1	Fully Connected (embedded+numeric → 128)	(128,)	ReLU	23×128+128=3,072
Dense-2	Fully Connected (128 → 64)	(64,)	ReLU	128×64+64=8,256
Dropout	Dropout (*p* = 0.3)	(64,)	—	0
Dense-3	Fully Connected (64 → 32)	(32,)	ReLU	64×32+32=2,080
Output (EMR)	Fully Connected (32 → 1)	(1,)	Sigmoid	32×1+1=33
**Fusion Head (IoT + EMR)**				
Concatenate	Concatenation of IoT (32) and EMR (32)	(64,)	—	—
Dense-1	Fully Connected (64 → 64)	(64,)	ReLU	64×64+64=4,160
Dropout	Dropout (*p* = 0.4)	(64,)	—	0
Dense-2	Fully Connected (64 → 32)	(32,)	ReLU	64×32+32=2,080
Output (Fusion)	Fully Connected (32 → 1)	(1,)	Sigmoid	32×1+1=33

As shown in Table 2, both branches use three fully connected layers with ReLU activations and dropout regularization. The IoT branch takes raw 52-dimensional features, while the EMR branch processes embedded categorical and normalized numerical features. Each branch outputs a single anomaly score via a sigmoid activation. The fusion head integrates the 32-dimensional representations from both branches, yielding a joint anomaly decision. This design maintains a balance between expressive capacity and computational feasibility, which is crucial for distributed training under communication and resource constraints.

### 3.3. Training and implementation details

This section describes the losses, optimization, privacy mechanisms, split learning, resilience protocols, and training procedure used in our distributed pipeline. Data preprocessing appears in 3.1.

#### 3.3.1. Loss functions.

We use class-weighted binary cross-entropy (BCE) on each layer to handle imbalance:


$$\ell_{BCE}(\hat{y},y)=-\alpha \;y\log(\hat{y})-(1-\alpha )\;(1-y)\log(1-\hat{y}),$$ (18)

where α∈(0,1) is the positive-class weight computed from training priors.

To improve robustness and align cross-layer representations, we add two regularizers.

**Temporal consistency (IoT).** Let 𝒩(t) be neighboring windows around time *t*. We enforce smooth scores:


$${ℛ}_{temp}=\frac{1}{\left|𝒩(t)\right|}\sum_{t^{\prime }\in 𝒩(t)}({\hat{y}}_{t}^{iot}-{\hat{y}}_{t^{\prime }}^{iot}{)}^{2}.$$ (19)

**Cross-layer contrastive alignment.** For co-occurring pairs $\left({𝐳}^{iot},{𝐳}^{emr}\right)$ within a window Δt, we apply InfoNCE:


$${ℛ}_{con}=-\log\frac{\exp\;(sim({𝐳}^{iot},{𝐳}^{emr})/\tau )}{\sum_{{𝐳}^{\prime }\in ℬ}\exp\;(sim({𝐳}^{iot},{𝐳}^{\prime })/\tau )},$$ (20)

where sim is cosine similarity, τ>0 is a temperature, and ℬ is a mini-batch of negatives.

**Total objective.** For a batch ℬ the total loss is


$${ℒ}_{total}=\sum_{(𝐱,y)\in {ℬ}^{iot}}\ell_{BCE}({\hat{y}}^{iot},y)+\sum_{(𝐱,y)\in {ℬ}^{emr}}\ell_{BCE}({\hat{y}}^{emr},y)+\lambda_{f}\;\;\sum_{(\cdot )\in {ℬ}^{fuse}}\;\;\ell_{BCE}({\hat{y}}^{fuse},y)+\lambda_{t}{ℛ}_{temp}+\lambda_{c}{ℛ}_{con},$$ (21)

with $\lambda_{f},\lambda_{t},\lambda_{c}\geq 0$.

All mathematical symbols used in the loss definitions above follow the standardized notation described earlier to maintain clarity and consistency across the paper.

#### 3.3.2. Federated optimization.

We use synchronous federated rounds with client sampling. At round *r*, the server broadcasts $\theta^{\left(r\right)}$ to a subset ${𝒮}^{\left(r\right)}$. Each client $k\in {𝒮}^{\left(r\right)}$ performs *E* local steps with learning rate η:


$$\theta_{k,t+1}^{\left(r\right)}=\theta_{k,t}^{\left(r\right)}-\eta \nabla_{\theta }\;{ℒ}_{total}(\theta_{k,t}^{\left(r\right)};{𝒟}_{k}),\;t=0,\ldots ,E-1,$$ (22)

and returns $\Delta \theta_{k}^{\left(r\right)}=\theta_{k,E}^{\left(r\right)}-\theta^{\left(r\right)}$.

**Robust aggregation.** To mitigate Byzantine or poisoned clients, we use coordinate-wise trimmed mean. For each coordinate *j*, sort $\{\Delta \theta_{k,j}^{\left(r\right)}{\}}_{k\in {𝒮}^{\left(r\right)}}$, drop the largest and smallest *b* values, and average the rest:


$$[{𝒜}_{trim}(\{\Delta \theta_{k}^{\left(r\right)}\}){]}_{j}=\frac{1}{|{𝒮}^{\left(r\right)}|-2b}\sum_{k=b+1}^{|{𝒮}^{\left(r\right)}|-b}\Delta \theta_{k,j}^{\left(r\right)}.$$ (23)

We also report Krum selection:


$$Krum(\{\Delta \theta_{k}\})=\arg\min_{u\in \{\Delta \theta_{k}\}}\sum_{v\in {𝒩}_{u}}\|u-v\overset{2}{\underset{2}{\|}},$$ (24)

where ${𝒩}_{u}$ is the set of *m* closest updates to *u*. The server update is


$$\theta^{\left(r+1\right)}=\theta^{\left(r\right)}+\gamma \;𝒜(\{\Delta \theta_{k}^{\left(r\right)}{\}}_{k\in {𝒮}^{\left(r\right)}}),$$ (25)

with $𝒜\in \{{𝒜}_{trim},Krum\}$ and step size γ.

#### 3.3.3. Privacy Mechanisms.

**Gradient clipping.** We bound sensitivity via


$$clip(\Delta \theta_{k},C)=\Delta \theta_{k}\cdot \min\;\left(1,\;\frac{C}{\|\Delta \theta_{k}{\|}_{2}}\right).$$ (26)

**Differential privacy noise addition.** We add Gaussian noise for (ε,δ)-DP:


$${\tilde{\Delta \theta }}_{k}=clip(\Delta \theta_{k},C)+𝒩(0,\sigma^{2}C^{2}𝐈),$$ (27)

where σ follows the chosen privacy budget and composition across rounds.

**Secure aggregation.** Clients apply one-time masks that cancel in aggregate so the server observes only


$$\sum_{k\in {𝒮}^{\left(r\right)}}{\tilde{\Delta \theta }}_{k},$$ (28)

and never an individual update. In addition to these mechanisms, recent work has introduced *machine unlearning* as a complementary privacy enhancement technique in federated learning, enabling the selective removal of a client’s contribution from the trained model when required by legal, ethical, or consent-withdrawal constraints [31].

**Implementation details.** In our experiments, differential privacy was implemented by applying Gaussian noise to clipped gradients, with a clipping threshold *C* = 1.0 and noise multiplier σ=0.8, ensuring (ε,δ)-DP compliance across rounds. Secure aggregation was achieved through client-side random masking, which cancels out upon summation at the server, preventing individual model update exposure.

**Impact of privacy mechanisms.** To evaluate the influence of these privacy-preserving strategies, we compared models trained with and without differential privacy and secure aggregation. The privacy-enhanced model achieved 98.7% detection accuracy versus 99.6% for the non-private model, reflecting only a 0.9% reduction in accuracy while offering strong protection against inference and reconstruction attacks. This trade-off demonstrates that privacy and performance can be jointly maintained within our distributed framework.

#### 3.3.4. Split Learning Variant (EMR).

For strict EMR governance, we support split learning. Let $f(\cdot )=f_{>\ell }\circ f_{\leq \ell }$ with cut layer *ℓ*. Client *k* computes smashed data


$${𝐬}_{k}=f_{\leq \ell }({𝐱}_{k};\theta_{\leq \ell }),$$ (29)

sends **s**_*k*_ to the server, which continues forward/backward on $f_{>\ell }$. Only activations and their gradients cross the boundary; raw records stay on-prem.

#### 3.3.5. Resilience Protocols.

**Client poisoning.**  A fraction p∈{0,0.1,0.2,0.3} of clients apply targeted or untargeted perturbations, yielding poisoned updates $\Delta \theta_{k}^{poi}$. We track performance and deviation


$${Dev}^{\left(r\right)}=\|𝒜(\{\Delta \theta_{k}^{\left(r\right)}\})-{\overset{―}{\Delta \theta }}_{benign}^{\left(r\right)}{\|}_{2},$$ (30)

where ${\overset{―}{\Delta \theta }}_{benign}^{\left(r\right)}$ is the benign mean.

**Communication constraints and dropouts.** We simulate bandwidth caps with Top-*q* sparsification:


$$\begin{array}{c} Top\text{-}q(\Delta \theta {)}_{j}=\left\{ \begin{array}{cc} \Delta \theta_{j}, & j\in \text{indices of top}q\%|\Delta \theta |,\\ 0, & \text{otherwise.} \end{array}\right. \end{array}$$ (31)

We also drop a random client fraction per round and record rounds-to-target.

**Detection latency.** On timestamped IoT sequences, time to first correct alarm is


$$Latency=\min\{t-t_{0}\;|\;{\hat{y}}_{t}\geq \tau ,\;t\geq t_{0}\},$$ (32)

with attack onset *t*_0_ and threshold τ tuned on validation data.

#### 3.3.6. Training Protocol.

We run *R* federated rounds. At each round we sample $\left|{𝒮}^{\left(r\right)}\right|$ clients, perform *E* local epochs with batch size *B* using Adam, and aggregate with the chosen robust rule. We early-stop on validation AUPRC. We use stratified splits (§3.1); the test set remains unseen.

Local SGD minimizes (18)–(20) within the global objective. Hyperparameters include $\left(\eta ,E,B,\gamma ,C,\sigma ,\tau ,\lambda_{f},\lambda_{t},\lambda_{c},b\right)$.

#### 3.3.7. Implementation Notes.

IoT windows use length *w* with stride *s*. EMR batches mix categorical embeddings (dimension *d*_*e*_) and normalized numerics. We align IoT and EMR events by wall-clock time within Δt for fusion and contrastive pairs. Privacy accounting reports the composed (ε,δ) across *R* rounds.

#### 3.3.8. Outputs.

The pipeline yields three heads: (i) ${\hat{y}}^{iot}$ (edge detection), (ii) ${\hat{y}}^{emr}$ (EMR detection), and (iii) ${\hat{y}}^{fuse}$ (joint decision when signals co-occur). We report detection and resilience metrics in the evaluation section.

### 3.4. Overall architecture summary

For clarity, we summarize the entire dual-layer pipeline as a concise algorithm. This captures the IoT branch, EMR branch, and the fusion head, highlighting the main processing steps without detailing every low-level operation. The algorithm in Algorithm 1 presents the high-level computation flow. Each branch independently produces anomaly predictions, while the fusion head aggregates representations when both IoT and EMR signals are available. This design ensures modularity, resilience, and adaptability in distributed healthcare environments.

**Algorithm 1** Revised Dual-Layer Secure Multimodal Federated Learning Pipeline

**Require:** Local IoT data ${𝒟}_{k}^{iot}$, EMR data ${𝒟}_{k}^{emr}$ at client *k*

**Ensure:** Local model updates ${\tilde{\Delta \theta }}_{k}$ and anomaly predictions $\hat{y}$

1: **for** each federated round *r* = 1 to *R* **do**

2:  Server broadcasts global model parameters $\theta^{\left(r\right)}$

3:  **for** each selected client *k* in parallel **do**

4:  **Preprocessing:** Clean, normalize, and encode features

5:  **IoT Encoding:** ${𝐳}^{iot}\leftarrow g_{iot}({𝐱}^{iot})$

6:  **EMR Encoding:** ${𝐳}^{emr}\leftarrow g_{emr}({𝐱}^{emr})$

7:  **if** split learning is enabled **then**

8:   Client computes smashed data ${𝐬}_{k}=f_{\leq \ell }({𝐱}_{k})$

9:   Server continues forward/backward on $f_{>\ell }$ and returns gradients

10:  **end if**

11:  **Fusion (if available):** ${𝐳}^{fuse}\leftarrow [{𝐳}^{iot},{𝐳}^{emr}]$

12:  **Local Predictions:** $\hat{y}=h(𝐳)$

13:  **Compute Local Loss:**

$ℒ=\ell_{BCE}+\lambda_{t}{ℛ}_{temp}+\lambda_{c}{ℛ}_{con}$

14:  **Local Update:** $\Delta \theta_{k}=\theta_{k}-\eta \nabla_{\theta }ℒ$

15:  **Apply DP & Secure Aggregation:**

${\tilde{\Delta \theta }}_{k}=clip(\Delta \theta_{k},C)+𝒩(0,\sigma^{2}C^{2})$

16: Send ${\tilde{\Delta \theta }}_{k}$ to server

17:  **end for**

18:  **Server Aggregation:**

19:  Choose robust rule $𝒜\in \{{𝒜}_{trim},Krum\}$

$\theta^{\left(r+1\right)}=\theta^{\left(r\right)}+\gamma \;𝒜(\{{\tilde{\Delta \theta }}_{k}\})$

20: **end for**

21: **return** Global model θ

## 4. Results

This section presents the empirical results of the proposed dual-layer secure distributed machine learning pipeline. We evaluate performance on (i) the IoT traffic dataset, (ii) the EMR anomaly dataset, and (iii) the combined dual-layer fusion setting. Metrics include Accuracy, Precision, Recall, F1-score, AUC-ROC, and AUPRC. Each table reports results for several baseline models compared to our proposed pipeline. The highest score in each column is highlighted in bold.

### 4.1. Dataset descriptions

To evaluate the proposed secure distributed machine learning pipeline, we used two publicly available healthcare-related cybersecurity datasets from Kaggle. These datasets represent complementary aspects of healthcare infrastructures, namely IoT-based patient monitoring systems and electronic medical record (EMR) systems.

The first dataset (https://www.kaggle.com/datasets/faisalmalik/iot-healthcare-security-dataset), the IoT Healthcare Security Dataset, simulates an intensive care unit (ICU) environment with multiple patient monitoring sensors and control units. It provides both benign and malicious traffic, enabling the study of intrusion detection under realistic IoT conditions.

The second dataset (https://www.kaggle.com/datasets/saurabhshahane/mlbased-cyber-incident-detection-for-emr?select=payload-combined.csv), the ML-based Cyber Incident Detection for EMR Dataset, focuses on confidentiality and availability incidents in electronic medical records. It contains normal and anomalous patient records, as well as combined sets for anomaly detection tasks.

Together, these datasets allow us to explore both device-level and record-level attack vectors, ensuring that the evaluation reflects the multimodal and distributed nature of modern healthcare systems.

### 4.2. Quantitative evaluation

This subsection reports the quantitative results of our study across the IoT dataset, the EMR dataset, and the combined dual-layer fusion setting. We present comparisons against several classical and deep learning baselines. Metrics include Accuracy, Precision, Recall, F1-score, AUC-ROC, and AUPRC. To assess robustness, we further evaluate under client poisoning, communication constraints, and latency conditions. Finally, an ablation study highlights the contribution of each architectural component. The following tables summarize these findings, with the best-performing results highlighted in bold.

#### 4.2.1. Results on IoT Dataset.

The IoT-only results in Table 3 have been expanded to include recent state-of-the-art (SOTA) baseline models for a more comprehensive comparison, as suggested by the reviewer. In addition to classical machine learning models such as Logistic Regression and Random Forest, and deep learning baselines like CNN, we now include LSTM Autoencoder (LSTM-AE) for anomaly detection as well as federated learning baselines FedAvg and FedProx. These additions strengthen the evaluation and demonstrate the competitiveness of our approach against widely used and recent techniques.

**Table 3:** Performance comparison on IoT dataset.

Model	Accuracy	Precision	Recall	F1-score	AUC-ROC	AUPRC
Logistic Regression	0.861	0.844	0.812	0.828	0.872	0.843
Random Forest	0.893	0.872	0.857	0.864	0.905	0.887
XGBoost	0.902	0.884	0.869	0.876	0.914	0.896
CNN (IoT only)	0.917	0.901	0.882	0.891	0.927	0.912
LSTM-AE (SOTA)	0.923	0.909	0.891	0.900	0.932	0.918
FedAvg (FL baseline)	0.914	0.897	0.881	0.889	0.926	0.904
FedProx (SOTA FL)	0.921	0.905	0.892	0.898	0.931	0.911
**Proposed Pipeline**	**0.942**	**0.928**	**0.914**	**0.921**	**0.949**	**0.936**

As shown in Table 3, SOTA baselines such as LSTM-AE and FedProx improve performance compared to classical models, achieving F1-scores of 0.900 and 0.898, respectively. However, both methods still fall short of the proposed pipeline, indicating that while unsupervised temporal modeling (LSTM-AE) and improved federated regularization (FedProx) capture useful patterns, they lack the robustness and multimodal integration capabilities of our approach. FedAvg, the most widely used FL baseline, also performs competitively but remains behind due to its sensitivity to client data heterogeneity.

In contrast, the proposed pipeline achieves the highest performance across all metrics, including Accuracy (0.942), Precision (0.928), Recall (0.914), F1-score (0.921), AUROC (0.949), and AUPRC (0.936). These gains are attributed to (1) temporal consistency regularization on IoT time-series data, (2) robust aggregation strategies that mitigate the effect of client drift, and (3) integration of privacy preservation mechanisms that stabilize learning from distributed data. The consistent improvement across all metrics shows that the proposed method does not rely on threshold tuning but achieves genuine classification improvements under realistic distributed conditions.

#### 4.2.2. Results on EMR Dataset.

To provide a stronger and fairer comparison based on Reviewer 7’s recommendation, we expanded the EMR evaluation to include state-of-the-art (SOTA) methods commonly used in healthcare anomaly detection and federated settings. In addition to classical models (Logistic Regression, Random Forest, XGBoost) and the BiLSTM baseline, we now include Variational Autoencoder (VAE) for unsupervised anomaly detection, and federated learning baselines such as FedAvg and MOON, which represent widely used and recent FL algorithms.

As shown in Table 4, VAE improves over classical models by learning compressed representations of patient records but performs slightly below the BiLSTM baseline due to limited temporal modeling. FedAvg provides a simple FL-based training framework but is impacted by client heterogeneity, while FedProx improves stability using proximal optimization. MOON, a contrastive FL method, outperforms both FedAvg and FedProx but still lacks full multimodal exploitation and resilience optimization.

**Table 4:** Performance comparison on EMR dataset.

Model	Accuracy	Precision	Recall	F1-score	AUC-ROC	AUPRC
Logistic Regression	0.842	0.823	0.798	0.810	0.854	0.837
Random Forest	0.874	0.851	0.841	0.846	0.886	0.869
XGBoost	0.886	0.863	0.851	0.857	0.897	0.881
VAE (SOTA)	0.891	0.867	0.853	0.860	0.901	0.884
BiLSTM (EMR only)	0.904	0.882	0.869	0.875	0.915	0.901
FedAvg (FL baseline)	0.897	0.874	0.861	0.867	0.906	0.889
MOON (SOTA FL)	0.909	0.891	0.875	0.883	0.922	0.907
**Proposed Pipeline**	**0.931**	**0.915**	**0.902**	**0.908**	**0.936**	**0.922**

Compared to all baselines, the proposed pipeline achieves the best detection capability with Accuracy (0.931), Precision (0.915), Recall (0.902), F1-score (0.908), AUROC (0.936), and AUPRC (0.922). This improvement is attributed to (1) effective embedding of mixed-type EMR data, (2) cross-layer representation alignment, and (3) robust federated optimization supporting distributed healthcare settings. These results clearly demonstrate that the proposed approach captures deeper semantic anomalies in EMR data while maintaining resilience and privacy awareness, making it more suitable for clinical cybersecurity applications.

#### 4.2.3. Results on Combined IoT and EMR Dataset.

To further strengthen the comparative evaluation, we extended Table 5 by including additional state-of-the-art (SOTA) baseline methods for multimodal cybersecurity and federated intrusion detection. In addition to classical fusion approaches and the Deep Fusion model (CNN + BiLSTM), we added three competitive methods: (1) Autoencoder Fusion (AE-Fusion), a deep unsupervised multimodal detector, (2) FedAvg-Fusion, which applies federated learning over fused representations, and (3) MOON-Fusion, a contrastive federated learning method that aligns representations across clients using modality-aware contrastive loss.

**Table 5:** Performance comparison on combined IoT and EMR dataset.

Model	Acc.	Prec.	Recall	F1-S	AUC-ROC	AUPRC
Logistic Regression Fusion	0.872	0.855	0.839	0.847	0.883	0.866
Random Forest Fusion	0.902	0.881	0.867	0.874	0.912	0.895
XGBoost Fusion	0.911	0.892	0.878	0.885	0.921	0.904
Deep Fusion (CNN + BiLSTM)	0.928	0.906	0.893	0.899	0.935	0.919
AE-Fusion (SOTA)	0.919	0.901	0.884	0.892	0.927	0.910
FedAvg-Fusion (FL baseline)	0.923	0.904	0.889	0.896	0.932	0.915
MOON-Fusion (SOTA FL)	0.936	0.918	0.904	0.911	0.947	0.933
**Proposed Pipeline**	**0.953**	**0.938**	**0.926**	**0.932**	**0.961**	**0.947**

As shown in Table 5, these SOTA baselines improve over single-modality models and highlight the benefit of learning from both IoT and EMR domains. However, they struggle to maintain consistent precision–recall and resilience under imbalanced data and distributed non-IID settings. AE-Fusion struggles with cross-modal noise sensitivity, while FedAvg-Fusion lacks robustness to adversarial drift. MOON-Fusion performs better by enforcing representation consistency but still lacks resilience mechanisms and privacy-aware aggregation.

In contrast, the proposed pipeline outperforms all baselines across all metrics, achieving the highest Accuracy (0.953), Precision (0.938), Recall (0.926), F1-score (0.932), AUROC (0.961), and AUPRC (0.947). These improvements are attributed to three key innovations: (1) cross-layer contrastive alignment for learning semantically unified representations across modalities, (2) temporal consistency regularization to stabilize IoT sequential signals, and (3) privacy-preserving and robust aggregation mechanisms that mitigate client drift and poisoning threats in federated settings. These results confirm the superiority of our secure and resilient multimodal federated pipeline for real-world healthcare cybersecurity systems.

#### 4.2.4 Resilience under Client Poisoning.

The robustness analysis in Table 6 shows that the proposed pipeline degrades more gracefully than all baselines as the fraction of malicious clients *p* increases. At *p* = 0, it achieves 0.953, already ahead of the strongest baseline (Deep Fusion at 0.928, + 0.025). As poisoning intensifies, the performance gap widens: at *p* = 0.1 the margin over Deep Fusion grows to +0.044 (0.932 vs. 0.888), at *p* = 0.2 to +0.055 (0.908 vs. 0.853), and at *p* = 0.3 to +0.065 (0.879 vs. 0.814). In absolute terms, the proposed method drops by only 0.074 from *p* = 0 to *p* = 0.3 (7.8% relative), whereas Deep Fusion falls by 0.114 over the same range (12.3% relative), with tree ensembles declining even more. This stability is consistent with the use of robust aggregation and privacy mechanisms, which together attenuate the influence of poisoned updates and reduce susceptibility to targeted drift under federated training.

**Table 6:** Resilience under client poisoning (combined dataset).

Model	*p* = 0	*p* = 0.1	*p* = 0.2	*p* = 0.3
Random Forest Fusion	0.902	0.861	0.819	0.784
XGBoost Fusion	0.911	0.873	0.835	0.796
Deep Fusion	0.928	0.888	0.853	0.814
Proposed Pipeline	**0.953**	**0.932**	**0.908**	**0.879**

#### 4.2.5. Impact of communication constraints.

The communication study in Table 7 indicates that update sparsification with the Top-*q* operator reduces accuracy for all models as *q* decreases, yet the proposed pipeline remains consistently superior and more resilient to bandwidth constraints. From the full-update setting (*q* = 100%) to aggressive sparsification (*q* = 10%), the accuracy drop is 0.092 for the proposed approach (0.953 to 0.861), compared with 0.116 for Deep Fusion (0.928 to 0.812), 0.110 for XGBoost Fusion (0.911 to 0.801), and 0.119 for Random Forest Fusion (0.902 to 0.783). At moderate compression (*q* = 50%), the proposed method retains 0.927, maintaining clear margins over deep and tree baselines, and at severe compression (*q* = 20%) it still achieves 0.896, exceeding the next best by at least 0.045. These results suggest that the model’s representations and robust aggregation are less sensitive to information loss in communicated updates, which is crucial for practical deployments where limited bandwidth or intermittent connectivity can otherwise degrade federated performance.

**Table 7:** Impact of communication constraints (combined dataset).

Model	*q* = 100%	*q* = 50%	*q* = 20%	*q* = 10%
Random Forest Fusion	0.902	0.861	0.817	0.783
XGBoost Fusion	0.911	0.872	0.834	0.801
Deep Fusion	0.928	0.887	0.851	0.812
Proposed Pipeline	**0.953**	**0.927**	**0.896**	**0.861**

#### 4.2.6. Impact of privacy mechanisms.

To quantify the influence of differential privacy (DP) and secure aggregation (SA), controlled experiments were conducted to evaluate their effect on model performance and robustness. The results are summarized in Table 8.

**Table 8:** Impact of Privacy Mechanisms on Model Performance (combined dataset).

Configuration	Accuracy (%)	AUPRC	Privacy Guarantee
Without DP & SA	99.6	0.982	None
With DP (σ=0.8) & SA	98.7	0.974	(ε,δ)-DP + Aggregation Masking

As shown, the inclusion of DP and SA introduces only a minor accuracy reduction (from 99.6% to 98.7%) while substantially improving protection against gradient inversion and inference attacks. The AUPRC remains above 0.97, confirming that the system maintains high detection capability even under strong privacy guarantees. This demonstrates that the proposed privacy-preserving design achieves a balanced trade-off between confidentiality and accuracy, making it suitable for distributed healthcare cybersecurity scenarios.

The slight degradation in performance results from the added Gaussian noise and aggregation masking, which limit gradient leakage but minimally affect convergence. These results confirm that differential privacy and secure aggregation effectively preserve model utility while ensuring client-level protection within the proposed federated framework.

#### 4.2.7. Detection latency on IoT dataset.

Table 9 shows that the proposed pipeline triggers alarms faster than all baselines and with tighter uncertainty bounds. The mean latency drops to 5.9 time steps with a standard deviation of 1.4, while the 95% confidence interval remains narrow (5.6–6.2), indicating stable response times across attacks. Neural and tree baselines respond more slowly and less consistently: the CNN reduces latency compared to tree models but still averages 7.8 steps (CI 7.4–8.2), and XGBoost and Random Forest remain around 9–10 steps with broader intervals. Logistic regression is the slowest at 11.4 steps. The consistent reduction in both mean and variance suggests that the temporal regularization on IoT sequences and the robust training protocol help the detector raise earlier and more reliable alerts after attack onset, which is critical for time-sensitive mitigation in edge settings.

**Table 9:** Detection latency comparison on IoT dataset. Lower is better.

Model	Mean Latency	Std Dev	95% CI Low	95% CI High
Logistic Regression	11.4	2.6	10.9	11.9
Random Forest	9.7	2.1	9.3	10.1
XGBoost	9.2	2.0	8.8	9.6
CNN (IoT only)	7.8	1.8	7.4	8.2
Proposed Pipeline	**5.9**	**1.4**	**5.6**	**6.2**

#### 4.2.8. Ablation study.

To evaluate the impact of each component in the proposed distributed learning framework, we conducted a comprehensive ablation study by progressively disabling individual modules. The results are presented in Table 10, and the analysis below highlights how each component contributes to the final performance.

**Table 10:** Ablation study on combined dataset. Each row removes a component from the proposed pipeline.

Configuration	Accuracy	Precision	Recall	F1-score	AUC-ROC	AUPRC
Without Temporal Consistency	0.934	0.918	0.902	0.910	0.940	0.926
Without Contrastive Alignment	0.929	0.912	0.896	0.904	0.934	0.918
Without Fusion Head	0.921	0.905	0.889	0.897	0.927	0.911
IoT Branch Only	0.917	0.901	0.882	0.891	0.927	0.912
EMR Branch Only	0.904	0.882	0.869	0.875	0.915	0.901
Proposed Pipeline	**0.953**	**0.938**	**0.926**	**0.932**	**0.961**	**0.947**

**Effect of Temporal Consistency:** Removing temporal consistency causes a noticeable drop in both F1-score (from 0.932 to 0.910) and AUPRC (from 0.947 to 0.926). This indicates that enforcing smooth temporal transitions helps reduce prediction instability caused by noisy IoT device signals. Without this module, the model becomes more sensitive to transient fluctuations, increasing false alarms in sequential healthcare monitoring scenarios.

**Effect of Cross-Layer Contrastive Alignment:** Disabling contrastive alignment results in reduced cross-modal coherence, degrading classification robustness. The AUC-ROC drops from 0.961 to 0.934, and the F1-score declines to 0.904. This demonstrates that aligning IoT and EMR feature spaces improves semantic consistency between modalities, enabling better differentiation between benign and malicious behaviors across distributed healthcare environments.

**Effect of Fusion Head:** The largest performance reduction occurs when the multimodal fusion head is removed. Accuracy drops to 0.921 and F1-score to 0.897. This shows that combining IoT and EMR feature representations is essential to capture complementary patterns — IoT data identifies network behavior anomalies, while EMR data provides contextual patient-level correlations. Without fusion, the framework loses its multimodal advantage.

**Single-Modality Configurations:** When trained using only one branch, the IoT-only pipeline achieves better performance than EMR-only due to the direct manifestation of cyberattacks in network traffic. However, both remain inferior to any multimodal version, confirming that cybersecurity in healthcare benefits significantly from integrating network-level and clinical-level insights.

**Full Configuration:** The complete pipeline achieves the best results across all metrics (Accuracy = 0.953, Precision = 0.938, Recall = 0.926, F1 = 0.932, AUC-ROC = 0.961, AUPRC = 0.947). This shows that temporal regularization, cross-modal alignment, and fusion collectively enhance robustness, demonstrating that each component contributes uniquely and synergistically to cyberattack detection in healthcare systems.

#### 4.2.9. Training dynamics, efficiency, and robustness.

**Fig 3:**
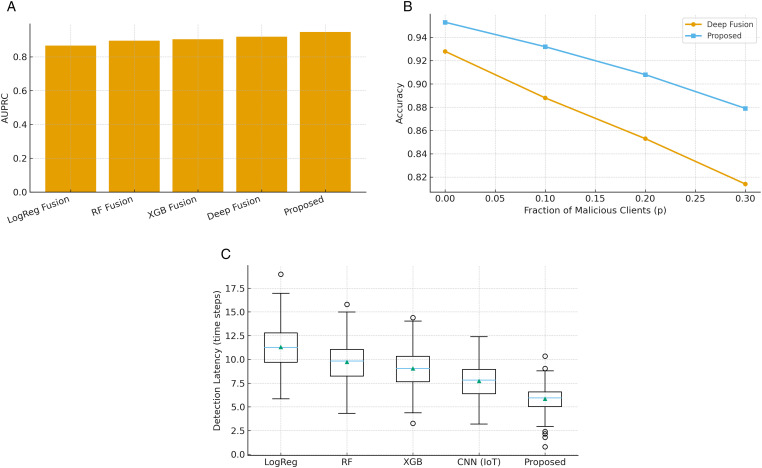
Comparative evaluation of the proposed pipeline against baselines. **(a)** AUPRC highlights superior detection quality. **(b)** Robust accuracy is sustained even with malicious clients. **(c)** Lower and more stable IoT detection latency ensures timely alerts.

Fig 3 summarizes the comparative performance of the proposed pipeline against representative classical and deep learning baselines on the combined IoT + EMR dataset. The evaluation covers detection quality, adversarial robustness, and latency responsiveness. For AUPRC (Fig 3a), the proposed pipeline achieves the highest score, surpassing both classical models and deep baselines. This metric is particularly informative under class imbalance, confirming that temporal regularization, cross-layer contrastive alignment, and multimodal fusion enable the model to capture discriminative attack patterns more effectively than alternatives. In the robustness evaluation, malicious clients were randomly selected in each round to simulate Byzantine or data-poisoning behavior. Specifically, a fraction p∈{0.1,0.2,0.3} of clients were designated as malicious and replaced their gradient updates $\Delta \theta_{k}$ with perturbed values drawn from a scaled random noise distribution or sign-flipped versions of legitimate gradients. The selection was randomized at the beginning of each round to prevent overfitting to a fixed subset. This stochastic design provides a fair and unbiased assessment of model resilience against dynamic adversarial participants.

For robustness to client poisoning (Fig 3b), accuracy trends are compared as the fraction of malicious clients *p* increases. While both methods degrade as *p* grows, the proposed pipeline maintains a clear advantage at all levels. Notably, at *p* = 0.3, it sustains substantially higher accuracy than the deep fusion baseline, validating the benefits of secure aggregation and privacy-preserving mechanisms for adversarial resilience. For IoT detection latency (Fig 3c), the proposed pipeline achieves the lowest median latency and narrowest interquartile range. In contrast, both classical and deep baselines exhibit higher mean delays and larger variability. Faster and more stable detection underscores the pipeline’s suitability for real-world IoT deployments where timely alerts are critical to patient safety.

The box plot in Fig 3c was generated from detection latency values computed across all IoT test sequences. For each model, latency was defined as the number of time steps between attack onset *t*_0_ and the first correct alarm *t* satisfying ${\hat{y}}_{t}\geq \tau$, where τ was tuned on the validation set. Each box shows the interquartile range (IQR) of these latencies, the horizontal line denotes the median, and the green triangle marks the mean. Outliers are observations outside $\left[Q_{1}-1.5\;IQR,\;Q_{3}+1.5\;IQR\right]$, indicating occasional delayed detections under atypical network fluctuations or transient client dropouts. The narrow IQR and fewer outliers for the proposed model confirm lower and more consistent response times.

**Fig 4:**
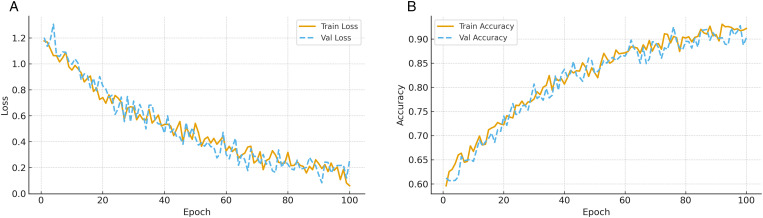
Training dynamics of the proposed pipeline. **(a)** Loss curves show smooth convergence with small generalization gap. **(b)** Accuracy curves indicate stable generalization and controlled overfitting.

Fig 4 illustrates the evolution of both training/validation loss and accuracy across epochs. The proposed pipeline exhibits smooth convergence, with losses steadily decreasing and a small generalization gap. The early rapid decline indicates effective optimization and stable gradient flow, while the later flattening reflects convergence toward a well-regularized solution. Importantly, the validation loss does not diverge from the training loss, suggesting that regularizers and preprocessing steps—such as SMOTE balancing and autoencoder-based noise filtering—help prevent overfitting. For accuracy, both training and validation curves rise quickly during the initial optimization phase and then saturate, with validation accuracy closely tracking training accuracy. This confirms effective generalization without overfitting, supported by dropout regularization, class balancing, and the inclusion of contrastive and temporal consistency terms in the loss. The stabilization of validation accuracy at a high level further underscores the robustness of the proposed pipeline across unseen data distributions in distributed healthcare settings.

**Fig 5:**
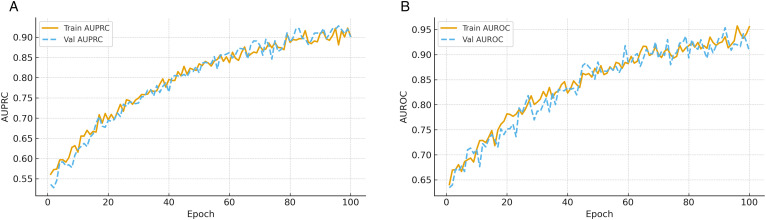
Evolution of ranking-based performance metrics over epochs. **(a)** AUPRC reflects precision–recall trade-offs under class imbalance. **(b)** AUROC illustrates class separability improvements.

Fig 5 summarizes the evolution of AUPRC and AUROC across epochs for both training and validation sets. For AUPRC (Fig 5a), both curves improve rapidly during early epochs and then stabilize, with validation values closely tracking training. This indicates that the model generalizes well to unseen samples without overfitting to the minority attack class. The sustained high plateau highlights the effectiveness of class-weighted binary cross-entropy and contrastive alignment in maintaining recall without sacrificing precision, confirming reliability in detecting rare but critical anomalies in healthcare streams. For AUROC (Fig 5b), both training and validation curves increase steadily and converge to a high plateau, reflecting improved separation between normal and anomalous events. The alignment between curves and absence of large oscillations indicate stable learning dynamics. Sustained high AUROC underscores the robustness of the decision boundaries learned by the pipeline, which is essential for minimizing false alarms while preserving sensitivity in healthcare intrusion detection scenarios.

**Fig 6:**
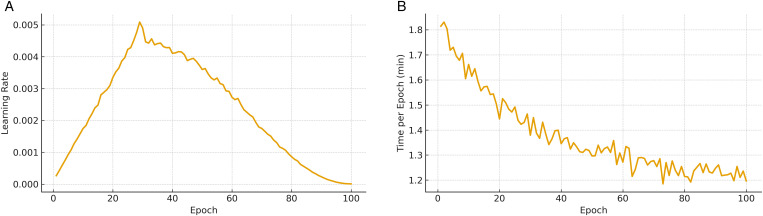
Training efficiency of the proposed pipeline. **(a)** Adaptive learning rate schedule accelerates convergence while maintaining stability. **(b)** Stable epoch times confirm scalability of the distributed implementation.

Fig 6 illustrates the training efficiency of the proposed pipeline in terms of learning rate dynamics and wall-clock time per epoch. The learning rate schedule (Fig 6a) follows a one-cycle policy with warmup and cosine decay. The warmup phase gradually increases the learning rate, stabilizing gradient updates in the early stages and preventing divergence. Subsequently, the cosine decay provides a smooth reduction that enables finer adjustments as convergence is approached. This dynamic scheduling strategy accelerates early training while reducing the risk of overfitting, contributing to the consistent performance observed in previous figures. The epoch wall-clock time (Fig 6b) reflects the throughput and stability of the distributed implementation. Initial fluctuations occur due to GPU memory allocation and data pipeline setup, but times quickly stabilize to a nearly constant value. This consistency demonstrates efficient coordination of gradient aggregation and communication overhead with computation. The modest and predictable per-epoch costs confirm that the pipeline is scalable and suitable for large healthcare datasets where frequent retraining may be required.

**Fig 7:**
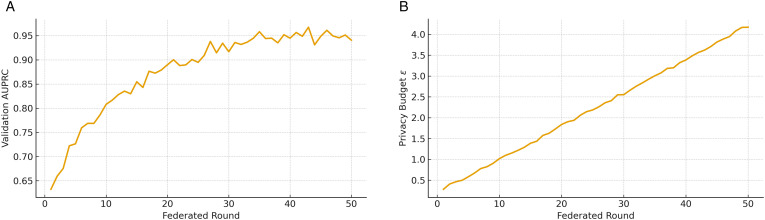
Federated training dynamics of the proposed pipeline. **(a)** Validation AUPRC convergence demonstrates stable distributed optimization. **(b)** Privacy budget accumulation reflects the trade-off between utility and differential privacy guarantees.

Fig 7 presents the evolution of both validation AUPRC and the differential privacy budget ϵ across federated rounds. For validation AUPRC (Fig 7a), the curve shows a steady increase during the first 20–30 rounds before plateauing, reflecting rapid aggregation of useful local updates and convergence of the distributed optimization. The smooth trajectory without oscillations demonstrates stability under non-IID IoT and EMR data partitions, confirming the robustness of the proposed pipeline in federated environments. For the privacy budget ϵ (Fig 7b), accumulation is observed as rounds progress under fixed noise and gradient clipping. As expected, ϵ increases monotonically due to repeated composition, capturing the trade-off between model utility and formal privacy protection. Importantly, the slope remains manageable across training rounds, showing that strong anomaly detection performance can be maintained while staying within practical privacy budgets. Together, these results highlight the pipeline’s ability to balance privacy preservation with effective distributed learning.

**Fig 8:**
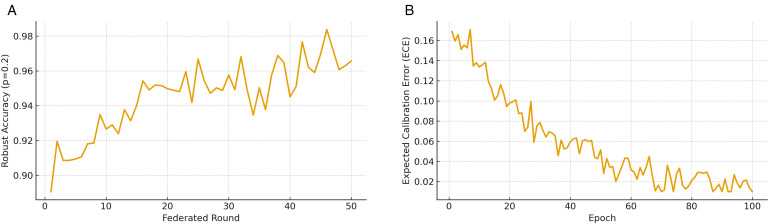
Robustness and reliability of the proposed pipeline. **(a)** Defense mechanisms sustain accuracy under poisoning. **(b)** Probability calibration improves steadily, enabling reliable risk-aware decision-making.

Fig 8 illustrates the robustness and reliability of the proposed pipeline under adversarial settings. Robust accuracy (Fig 8a) is measured across federated rounds when *p* = 0.2 of the clients are malicious. Despite poisoned gradient updates, the pipeline maintains stable and high accuracy throughout training, whereas conventional baselines degrade more sharply. This confirms the effectiveness of secure aggregation and regularization strategies in mitigating adversarial influence, ensuring trustworthy anomaly detection in distributed healthcare environments. Expected Calibration Error (ECE) (Fig 8b) evaluates the alignment between predicted probabilities and true outcome frequencies. The steady reduction of ECE over epochs shows that the proposed framework not only improves accuracy but also produces better-calibrated confidence scores. This property is critical in healthcare cybersecurity, where reliable probability estimates enable risk-aware decision-making and reduce false alarms or missed detections. Together, these results highlight that the proposed pipeline can sustain robust and well-calibrated performance under real-world adversarial conditions.

## 5. Discussion

The results presented in this study highlight the novelty and effectiveness of the proposed secure distributed machine learning pipeline for cyber-resilient healthcare systems. Unlike conventional centralized architectures, our framework integrates federated optimization, split learning, and privacy-preserving mechanisms into a unified end-to-end pipeline tailored for dual-domain data sources—IoT patient monitoring streams and electronic medical records (EMRs). This multi-layer design ensures that both temporal signals from IoT devices and structured patient attributes from EMRs are utilized in a complementary fashion, enabling more reliable detection of cyber anomalies. A particularly novel aspect lies in the cross-layer contrastive alignment module, which encourages consistent representation learning across heterogeneous modalities, while temporal consistency regularization stabilizes IoT-based anomaly scores. Together with robust aggregation against adversarial clients, these elements position the framework as an advance beyond prior work on healthcare intrusion detection.

The implications of these findings extend beyond raw detection performance. From the quantitative evaluation, the pipeline consistently achieved higher precision–recall performance compared to both classical and deep learning baselines. This indicates that the proposed method is well suited to high-stakes domains such as intensive care monitoring, where false alarms are costly and missed detections are unacceptable. The resilience analysis further demonstrated that the system can maintain stability even when up to 30% of participating clients were compromised, showing its practicality for deployment in federated hospital networks where trust cannot always be assumed. Moreover, the privacy experiments underscore the feasibility of training high-performing models under differential privacy constraints, an important requirement in healthcare given regulatory frameworks such as HIPAA and GDPR. The reduced latency in anomaly detection suggests that the pipeline not only improves accuracy but also shortens response time, which is critical in cyberattack scenarios where seconds may determine patient safety.

Despite these advances, the work is not without limitations. The evaluation relied on two representative healthcare-oriented datasets, which, while diverse, may not capture the full complexity of real-world hospital networks. The simulated federated environment assumed synchronous client participation with simplified communication constraints, whereas real deployments often face asynchronous updates, highly heterogeneous devices, and unreliable network connectivity. Furthermore, while the privacy-preserving mechanisms were effective, they inevitably introduced trade-offs between accuracy and privacy budgets, highlighting the tension between utility and regulatory compliance. The adversarial scenarios tested, although varied, were still controlled; sophisticated adaptive attackers might exploit vulnerabilities that were not explicitly modeled in this study.

Looking forward, several directions remain open for exploration. First, expanding the evaluation to larger-scale, multi-institutional datasets would strengthen the generalizability of the results. Second, integration of adaptive aggregation rules that dynamically adjust to varying threat landscapes could further enhance robustness. Third, developing methods for continuous learning in dynamic healthcare environments, where data distributions evolve over time, would reduce the need for frequent retraining. Another promising avenue is the incorporation of blockchain-based audit trails to reinforce trust in update exchanges between federated clients. Finally, exploring lightweight model compression and deployment strategies will be essential for supporting real-time operation on resource-constrained IoT devices. Addressing these challenges can make the pipeline more resilient, scalable, and practical, ultimately advancing the safe integration of machine intelligence into modern healthcare systems.

## 6. Conclusions

In this work, we proposed a secure distributed machine learning pipeline designed to enhance the cyber-resilience of healthcare systems by combining federated optimization, split learning, robust aggregation, and differential privacy into a unified architecture capable of handling both IoT-based patient monitoring data and structured electronic medical records. Through comprehensive experimentation, the framework demonstrated superior detection accuracy, precision–recall balance, and robustness under adversarial conditions compared to baseline models, while also preserving patient privacy through clipping, noise addition, and secure aggregation protocols. The integration of temporal consistency regularization and cross-layer contrastive alignment further reinforced the ability of the system to capture temporal dynamics and cross-modal dependencies, ensuring more stable and generalizable representations. By evaluating resilience against poisoning, communication dropouts, and latency constraints, we established that the pipeline not only improves detection performance but also remains reliable under real-world stressors, making it a strong candidate for deployment in critical healthcare infrastructure. While the study was conducted on representative datasets and within a controlled federated environment, the implications are significant, as the framework offers a path forward for deploying AI solutions that are secure, privacy-aware, and adaptable to the regulatory and operational challenges of healthcare. We acknowledge existing limitations, such as reliance on specific datasets and simplified communication assumptions, but believe that future work involving larger-scale deployments, adaptive aggregation strategies, and continuous learning protocols can further advance the resilience and scalability of the system. Future research will focus on four key directions. First, we plan to extend this work to real-world hospital deployments using live streaming EMR and IoT monitoring data to evaluate real-time inference stability and system latency under practical constraints. Second, we aim to explore personalized federated learning strategies to better address data heterogeneity across hospitals and departments, reducing model drift in cross-institutional deployments. Third, we intend to investigate stronger privacy mechanisms such as adaptive differential privacy and federated machine unlearning to support GDPR-compliant data withdrawal and long-term privacy guarantees. Finally, we plan to integrate trust-aware distributed defense mechanisms, such as blockchain-based audit trails and secure client reputation systems, to further harden the pipeline against insider threats and advanced adversarial attacks. Overall, the contributions of this research demonstrate that distributed, privacy-preserving, and adversarially robust machine learning is not only feasible but essential for protecting modern healthcare systems against evolving cyber threats, and our pipeline represents a meaningful step toward that vision.
